# Investigation of the Degradation Mechanism of the Tensile Mechanical Properties of Sandstone under the Corrosion of Various pH Solutions

**DOI:** 10.3390/ma16196536

**Published:** 2023-10-02

**Authors:** Luyi Huang, Hang Lin, Ping Cao, Qingxiong Zhao, Yongkang Pang, Weixun Yong

**Affiliations:** School of Resources and Safety Engineering, Central South University, Changsha 410083, China

**Keywords:** chemical corrosion, brazilian splitting, acoustic emission, damage evolution

## Abstract

The research aimed to examine the impact of different pH solutions on the tensile mechanical properties of saturated and natural sandstone specimens. The study utilized the WHY-300/10 microcomputer-controlled pressure testing machine to conduct Brazil splitting tests and employed acoustic emission and local dynamic strain testing techniques. The results indicated the tensile strength and split tensile modulus of the sandstone specimens gradually decreased with the polarisation of the solution pH, and the acoustic emission signal ring number monitoring values showed an increasing trend. The pH of the soaking solution followed an exponential decay pattern over time, eventually tending towards weak alkalinity. A new damage variable based on the cumulative ring count after chemical corrosion was defined to indirectly analyze the degree of corrosion degradation. It was discovered that in acidic or alkaline environments, the internal crystals of the rock are dissolved, weakening the mineral interconnections and causing a deterioration in tensile stress and strength. These findings can provide valuable insights for ensuring the safety and stability of the Denglou Mountain Tunnel in Yunnan Province.

## 1. Introduction

The rock-endowed water chemistry environment is complex and variable. Acid rain zones often occur in Southwest China, and the Ordos Basin is an alkaline rock-forming environment. Under long-term chemical dissolution and water flow, the rock body is corroded to produce microfissures, pores, joints and holes. Subsequently, destructive changes in the microstructure occur, leading to a reduction in the bearing capacity and stability of the rock mass. In actual working conditions, the Yuntai Mountain Tunnel in Henan Province has an acidic environment caused by water infiltration, which poses a great threat to the stability of the project. The Yangtze River Bridge support materials were partially corroded by the temperature of the Yangtze River and the acidic solution. Inadequate knowledge of rock chemical erosion leads to negligence and poor decision-making on engineering stability. Therefore, for the continuous safe operation of rock engineering, rock stability under rock chemical solution deterioration provides a theoretical guarantee. Hutchinson et al. [1] used sulphuric acid, hydrochloric acid, nitric acid solution and neutral salt solution to simulate the experimental study of acid rain corrosion on limestone. Yue et al. [2] used Na_2_SO_4_ and NaHCO_3_ solutions with different pH values to analyze the rock damage mechanism of water chemical corrosion. Numerous research results have shown that water and water chemistry are key factors in rock degradation and engineering instability under natural conditions.

Scholars have carried out damage mechanism studies on rocks under chemical solution corrosion. The results show that the chemical corrosion mechanism is affected by a variety of factors, such as the solution concentration [3,4], the composition of the solution substance [5], the mineral composition [6,7], the pH value of the solution [8], the immersion time [9,10] and other factors. There is a discernible pattern in the deterioration mechanism of chemical corrosion in rocks, with the pH value of the solution playing a significant role in the evolution of damage and the mechanical properties of rocks. Table 1 lists some of the research perspectives in the field of chemical corrosion. As seen from previous chemical corrosion research results, most of the water chemical corrosion damage studies are based on a single acidic solution and erosion ions. The research results on acid corrosion with acid rain as the research background have been abundant, but the research on alkali corrosion with seawater as the research background is insufficient.

In addition, rocks after chemical erosion may still be damaged by external loading. Currently, there is abundant stress-resistant chemical characterization under chemical erosion [18,19,20]. However, the tensile mechanical properties under chemical erosion conditions are understudied. In conclusion, there is a lack of comprehensive research on chemical erosion and tensile damage. In underground rock engineering, rock excavation forms a free surface, and rock damage around the free surface is mainly of the tensile damage mode. The research on the tensile mechanical properties of rocks has achieved many remarkable results. Scholars have conducted relevant studies on Brazilian splitting by combining acoustic emission [21], scattering [22,23], strain testing [24] and other technologies. In view of the lithology and anisotropy of the rock [25,26,27], the damage modes, mechanical properties and strength characteristics were investigated [28,29]. Therefore, the study of the tensile mechanical properties of rocks in chemical solutions is useful for guiding engineering. In this study, uniaxial tensile tests were carried out on Brazilian split sandstone specimens immersed in chemical solutions of different pH values.

## 2. Test Materials and Test Methods

### 2.1. Petrographic Study

**Figure 1:**
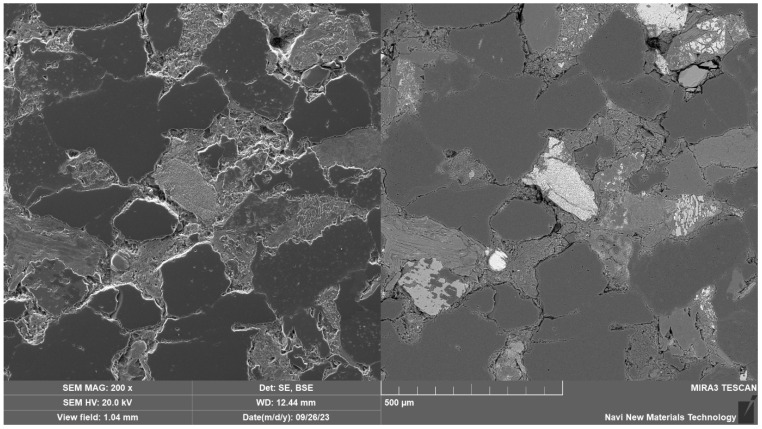
Electron scanning microscope image of the sample.

The rock mass of the test was sandstone, and the mineral composition was identified as a purplish-red, greyish feldspathic quartz medium sandstone. The sandstone is of massive formation, internally supported by grains in point-line contact. The components are in contact cementation with each other and the cement is calcite, which is a carbonate. The particle size of the granular material is about 0.26–0.5 mm, and the density is in the range of 2.60~2.63 g/cm^3^. The rounding is sub-prismatic-sub-rounded, and the sorting is medium. It mainly consists of feldspar, calcite and quartz, with about 30% feldspar, 35% quartz and 35% calcite particles. The structural image is shown in Figure 1:

### 2.2. Sample Preparation

The sandstone specimens for this test were taken from the middle of the left-line construction section of the Dengloushan Tunnel in Yunnan Province. To obtain good integrity and homogeneity of the test results, sandstone cores were drilled from the same rock mass in the vertical lamination direction. Then, sandstone cores were machined into standard Brazilian disc sandstone rock samples. The diameter was 50 ± 2 mm, the thickness was 25 ± 1 mm and the height-to-diameter ratio was 1:2.

### 2.3. Introduction of Specimen Equipment

**Figure 2:**
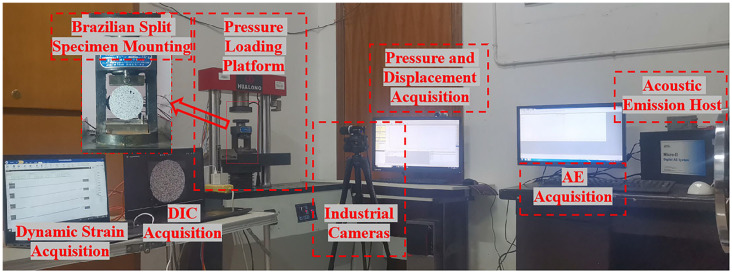
Test equipment.

The pH value of the chemical solution was measured by a Shanghai Leimagnet PHS-3C acidity meter with an accuracy of 0.01 and a range of 0 to 14. A wave velocity test was performed by HS-YS4A rock acoustic wave parameter tester. A DH5981 distributed network dynamic signal test and analysis system was used to collect and analyze the dynamic data. As shown in Figure 2, the Brazilian splitting test was completed on the WHY-300/10 microcomputer-controlled pressure testing machine of Central South University. The maximum test force was 300 kN and the loading rate was 200 N/s, using stress-controlled loading.

The acoustic emission signaling device is an 8-channel Micro-IIExpree Digital AE System manufactured by Physical Acoustics Inc. in the United States. Firstly, the petroleum jelly was uniformly applied to the contact area of the AE probe and the sample. Afterwards, the contact area between the AE probe and the sample was made by uniformly applying petroleum jelly during loading. And fixed with specialized acoustic emission probe magnetic iron sheets to prevent poor contact during testing. Adequate coupling between the AE probe and the rock sample was ensured. The parameters are shown in Table 2.

The Digital Impact Casting (DIC) technique can obtain the displacement and strain field characteristics of the specimen surface and is widely used in rock mechanics tests. The photos initially taken by the CCD camera were transferred to the scattering processing GOM software. By adjusting the image quality, the evolution of the displacement and strain fields of the specimen during the test can be obtained.

### 2.4. Pre-Treatment of Specimen

**Figure 3:**
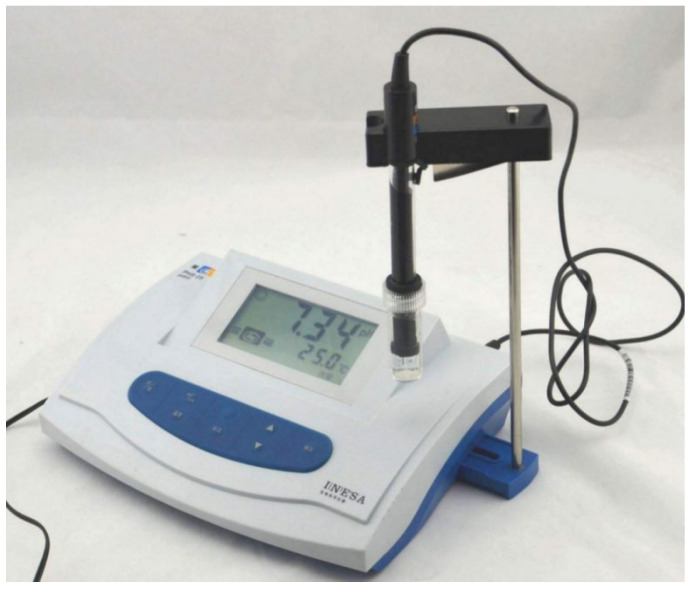
Specimen immersion.

Due to the complex and variable tunnel water chemistry environment, coupled with the long and slow water-rock interaction time. In order to observe the chemical corrosion changes in a short period of time without departing from the actual working conditions, this test used the Yunnan Dengloushan Mountain Tunnel site solution (pH = 7.68) as the substrate fluid. The basal solution was added with hydrochloric acid and sodium hydroxide solution to formulate chemical solutions with different respective pH values. The solution pH values were set into four gradients of 2, 5, 7.68 and 11, plus the natural state for a total of five working conditions. The configuration process was carried out by titration and the solution pH during titration was tested using an acidometer. Figure 3 below shows the pH audiometer.

Before the solution treatment, the wave velocity tester was used to select the target rock samples. The specimens were dried for 24 h in an oven at 105 °C. The mass of the specimen tended to change gently with the drying time and the rock specimen reached the drying state. At this point, the saturated sandstone samples were immersed in aqueous chemical solutions by vacuum pump method. The pH of each immersion solution was dynamically tested and recorded by acidimeter during the immersion process. When the solution pH tended to stabilize, it was considered to meet the specimen immersion time requirements. The specimen immersion time was 85 h. Fifteen sandstone samples were divided into 5 groups, each group of 3 specimens, the specimen parameter table is shown in Table 3. To obtain the internal acoustic emission characteristics of sandstone during the destruction process of Brazilian cleavage, the acoustic emission probe iron sheet was arranged on the surface of the rock samples. At the same time, to obtain the change in strain at the damaged surface, the strain gauges were pasted at the positions of 1/2D, 1/4D and 2/3D away from the top of the specimens. The other side was treated with black and white paint to obtain a random scatter collection surface.

### 2.5. Test Loading Process and Method

**Figure 4:**
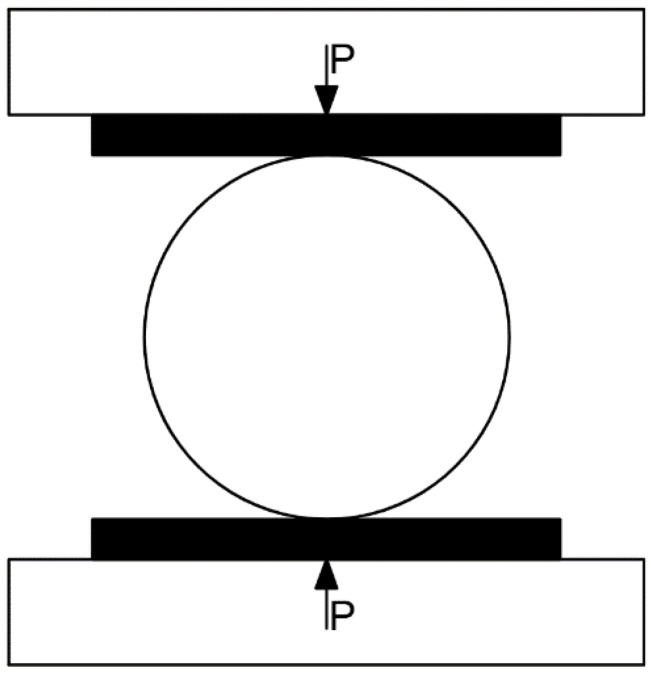
Schematic diagram of Brazilian splitting test.

There are four Brazilian splitting test methods [30,31,32]. However, the upper and lower curved steel plates (ISRM) are very harsh on specimen size. On the other hand, the “steel plate–thin iron bar–specimen” method (domestic rock mechanics test specification) has an effect on the acoustic emission test with the thin iron wire [33]. In this test, the direct pressure method adopted by Liu et al. [34] was chosen, which is also commonly used in mining sector specifications [35]. In addition, He et al. [36] showed the rock tensile strength values obtained from direct and arc loading are very close to each other. The schematic diagram of the platform method Brazilian splitting test is shown in Figure 4.

In the experimental process, the pressure testing machine was force-controlled, an initial load of 0.2 kN was applied before the test and the test loading rate was 0.2 kN/s. During the test, press loading, acoustic emission, dynamic strain collection (5 kHz) and industrial camera acquisition frequency (20 Hz) were synchronously switched on during the test. The load-displacement, acoustic emission signals, tensile strains at different locations and scattered speckle image data were collected during the experimental loading process, respectively.

## 3. Results and Discussion

### 3.1. Destruction Pattern

**Figure 5:**
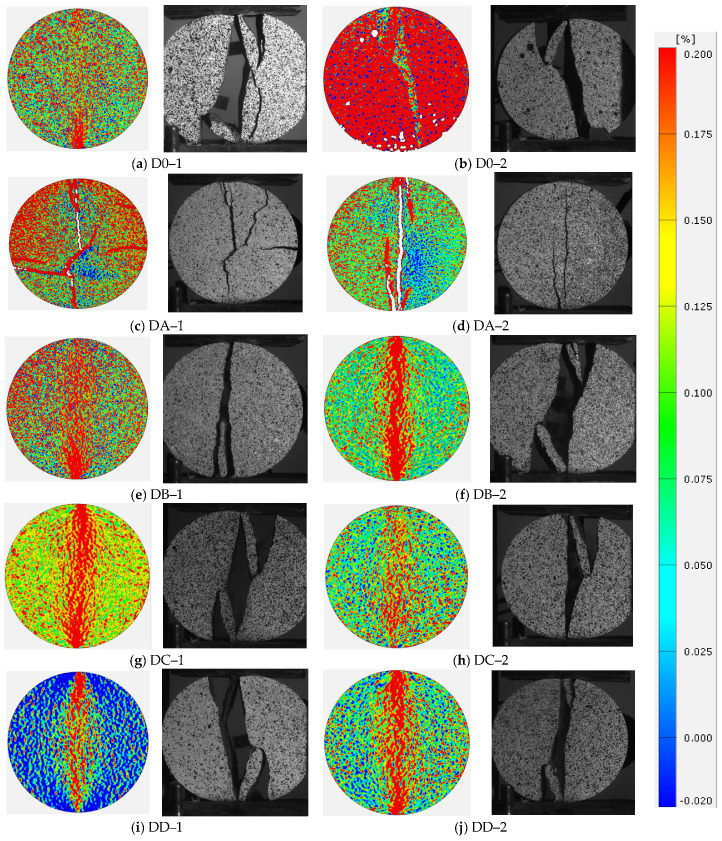
Critical damage maximum principal strain cloud and final damage scatter plot.

From Figure 5, the damage pattern is mainly tensile, with the rock samples mainly damaged in tension and ruptured at the upper and lower stress locations. The splitting surface roughly passes through the plane where the loading center and the center of the disc are located. Microfractures arise from the upper and lower loading ends, extending into each other until penetrating into macrofractures.

However, the damage extension of the specimens after immersion in different pH solutions varies. The bottom or top of the specimens appeared to exhibit varying degrees of fragmentation behavior. In addition to the primary cleavage in the diameter direction, secondary cleavage occurred at the bottom and end of the specimen loading. The rock samples were partially dislodged, and the internal cracking surface of the samples was uneven and undulating after splitting. This is mainly due to the internal defects of the rock samples, which affects the development of destructive cracks. The fracture surfaces of some rock samples deviated from the plane in which the loading baseline was located. Secondary fracture features were formed. In addition, the magnitude of local strains around the tensile cracks varied before the rock samples were damaged. Therefore, it can be concluded that the action of different pH solutions will have different deterioration effects on rock samples.

The damage region was enlarged, and the damage mode of the specimen at this time was tensile damage or mixed tensile and shear damage [37,38]. This damage pattern is consistent with the results obtained by Zhang et al. [39]. The damage pattern was lower compared to the damage pattern presented after 30 days of immersion. This is due to the shorter soaking time. In this research, the specimens have fewer crushed pieces of damage, and the soaking time can be increased for subsequent tests.

### 3.2. Mechanical Properties

#### 3.2.1. Displacement-Load Curve

**Figure 6:**
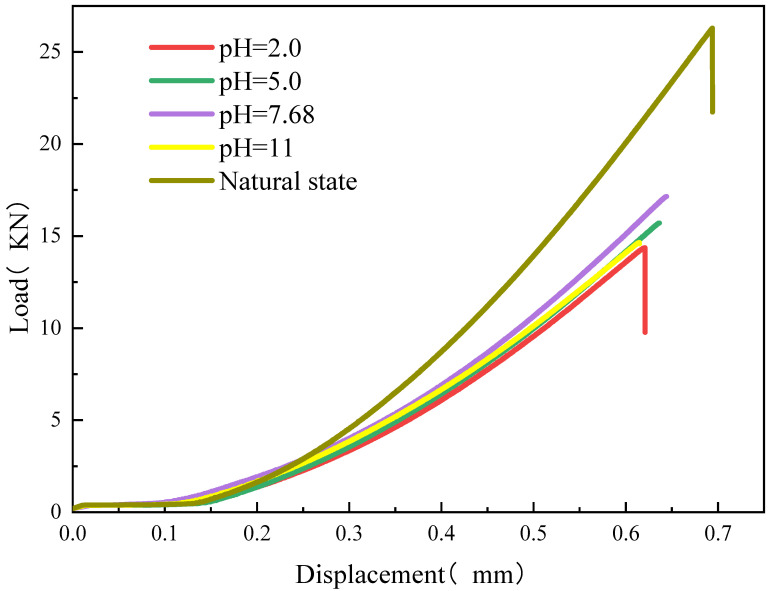
Displacement-load curve.

From the Figure 6, the overall trend and characteristics of the displacement-load curves are the same. The sandstone displacement-load curves are roughly divided into compression-density stage, elasticity stage and damage stage. Compared with the typical uniaxial compressive stress-strain curve, the rock sample does not have an obvious yielding stage. In the initial compaction stage, the curve exhibits an upward concave phenomenon. This stage is mainly the local pressure-tight deformation of the pressure plate and platform in contact with the specimen surface. Moreover, the contact area between the loading platform and the specimen increases as the load increases. As the load increases, the load and displacement are linearly related, showing the elastic characteristics of the material. When the load is gradually increased to the peak load, the specimen is suddenly destroyed. A brief post-peak phase can be seen in some of the curves, but the post-peak load drop curve falls vertically, becoming almost parallel to the coordinate axis. At this point, the sandstone specimen instantly splits and is destroyed, and some of the rock chips are splattered. This is due to the high brittleness index of the sandstone in the selected area, which shows typical brittle damage.

It is different in the load-deformation curves of rock samples under different pH immersions, mainly in the slope of the curves and the peak rock damage. Compared with the peak rock load damage in the natural state, the peak rock load damage of the rock samples after chemical corrosion was reduced. The higher the pH was, the smaller the peak load damage was. At pH = 2, 5, 7.68 and 11, the peak loads of the specimens after 85 h of corrosion were 14.36, 15.71, 17.16 and 14.66 kN, respectively. The peak stresses in the chemically corroded solutions were reduced by about 45%, 40%, 35% and 44%, respectively, as compared with that of the natural state with a peak load. The variation of peak axial displacement is much less for different pH values of chemical corrosion solutions, which was around 0.63 mm. The peak stress after acid and alkali corrosion shows a decreasing trend in good agreement with the results of Huang et al. [40].

#### 3.2.2. Analysis of Tensile Mechanical Parameters

(1) Tensile strength

According to the electromechanics calculation method in the ISRM standard method, the tensile strength was calculated based on the stress state at the center point of the disc. The tensile strength formula of the rock was calculated as follows:

(1) $$\sigma_{t}=\frac{2P_{\max}}{\pi DL}$$

where $\sigma_{t}$ is the Brazilian splitting tensile strength (MPa), *P_max_* is the peak load (N), *D* is the diameter of the sample (mm) and *L* is the thickness of the sample (mm).

**Figure 7:**
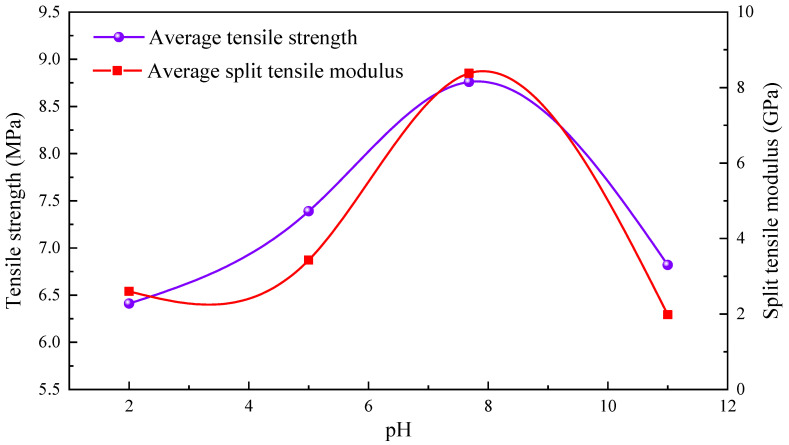
Tensile strength and splitting modulus.

The curves of tensile strength of the specimens after immersion in different pH solutions as a function of the pH of the immersion solution are shown in Figure 7. The results show a trend of weak alkali (pH = 7.68) > weak acid (pH = 5) > strong alkali (pH = 11) > strong acid (pH = 2). The greatest deterioration was in the rock samples after immersion in the chemical solution of strong acid (pH = 2), where the decrease in tensile strength was 43.62%. Furthermore, when compared to the natural state, the tensile strength of the rock decreased by 40.02%, 35% and 22.96% for sandstones with a pH of 11, 5 and 7.68, respectively. Due to the non-homogeneity of the rock material, the tensile strength of each tested rock sample has a certain degree of dispersion. However, the average tensile strength increases with the increase of the pH of the soaking solution. When the pH of the solution exceeds a certain threshold, the tensile strength of the rock samples decreases with the increase of the pH of the soaking solution. The threshold value depends mainly on the conditions under which the internal constituents of the sandstone react with the ions and other substances inside the solution.

The trend of decrease in tensile strength during corrosion in acidic and alkaline solutions is in agreement with the results of Parvizpour et al. [41], Dubey and Singh [42] and Fu et al. [43]. Overall, the reduction in intensity under acidic conditions was greater than that under alkaline conditions.

(2) Splitting modulus

The splitting tensile modulus depicts the relationship between deformation and load during the rock-splitting loading process. There are two methods for computing this modulus: (1) Stick strain gauges at the center perpendicular to the loading direction and solve for the tensile modulus of the rock by using the measured strain values [44,45]; (2) Yu [46] proposed a magnitude analysis method to define the splitting modulus. In this method, the vertical displacement curve is divided by the value of the rock’s radial surface (P/Sm), while the horizontal coordinate value is divided by the diameter of the specimen (Disp/D). The resulting curve has the same shape as the load-vertical displacement curve, and the slope of the straight line segment represents the modulus of cleavage. In this study, the magnitude analysis method is utilized to calculate the splitting modulus of sandstone.

As shown in Figure 7, the average value of instantaneous splitting modulus varies with the pH of the immersion solution at different pHs of the chemical solution. The change curves of splitting modulus and rock tensile strength have the same trend. The split tensile modulus of the rock samples firstly increases with the increase of the pH of the soaking solution and then decreases with the increase of the pH of the soaking solution when the pH of the solution exceeds a certain threshold value.

(3) Instantaneous splitting modulus

**Figure 8:**
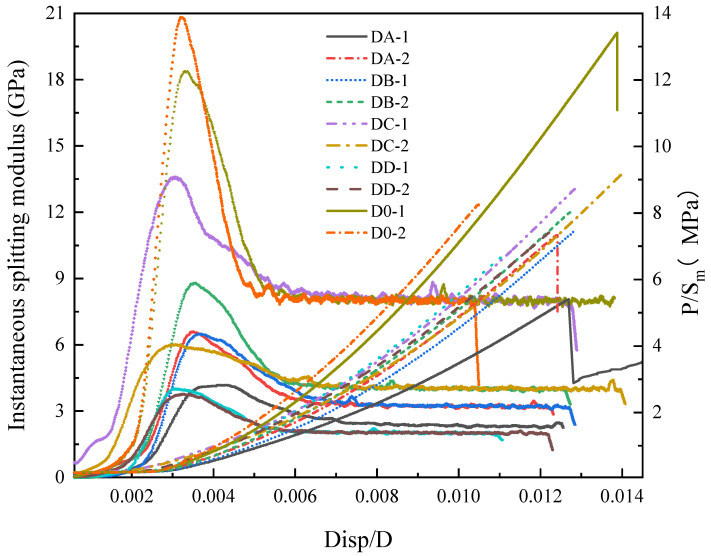
Splitting modulus.

To determine the eigenvalue points of each stage, the instantaneous splitting modulus was introduced. The differential value of the curve under Disp/D is taken as the value of the instantaneous splitting modulus. The curve of the instantaneous splitting modulus variation with Disp/D is shown in Figure 8 below.

**Figure 9:**
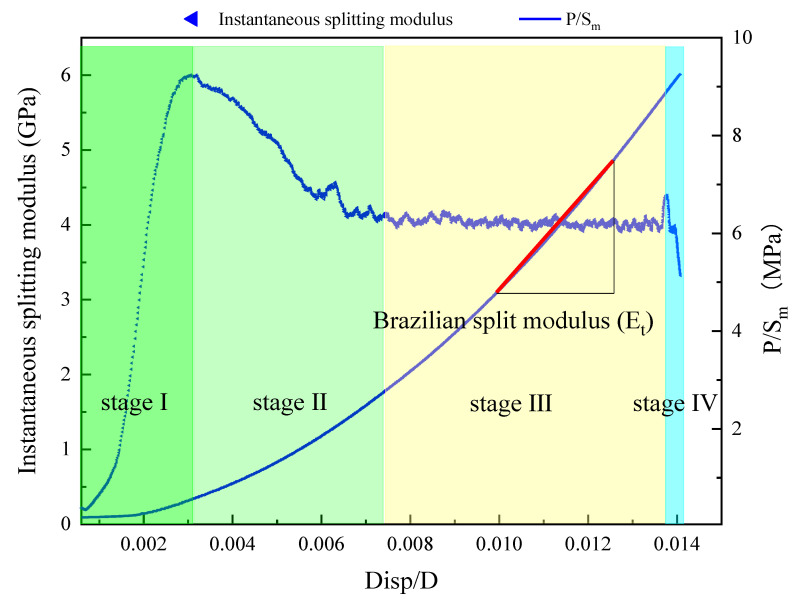
Displacement-instantaneous splitting modulus.

As depicted in Figure 9, the instantaneous splitting modulus initially increases and then decreases with the loading displacement, ultimately stabilizing. After a period of stabilization, the instantaneous splitting modulus experiences a rapid decrease. At the beginning of this stage, the instantaneous modulus first increases and then decreases, reaching a maximum value. This stage is primarily influenced by the loading process of the testing machine. When the load is small, the loading head gravity and the impact of hydraulic loading cause the instantaneous load to be elevated. At this stage, the dense compression is instantaneously observed. Due to the original inertia of the rock samples, deformation is minimal, leading to a rapid increase in the instantaneous splitting modulus. According to the load-displacement-load curve and the instantaneous splitting modulus change curve, the beginning and end points of the instantaneous elastic modulus invariant section can be obtained. The compression closure point at the end of the compression stage is the start point, and the crack opening point at the end of the elastic stage is the endpoint.

**Figure 10:**
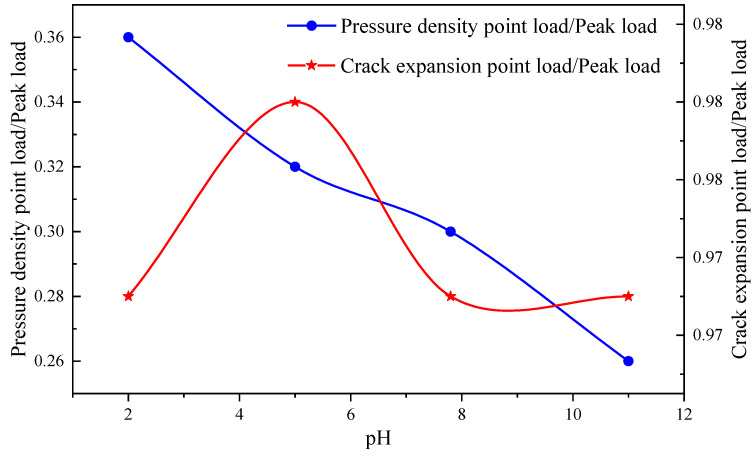
Compression density point, crack extension point and peak load.

From Figure 10, the solution pH affects the pressure-tightening point of sandstone Brazilian splitting. The pressure-tightening point to peak load ratio of sandstone gets smaller as the pH increases. However, this compaction load is between 22% and 36% of the peak load. This result is consistent with the theoretical results of stress threshold prediction for the compaction point in uniaxial compressive stress-strain curves obtained from the literature. However, the crack extension point load corresponding to the end of the elastic phase is about 98% of the peak load. The result differs from the theoretical value of the crack extension stress threshold prediction in the stress-strain curve of uniaxial compression. This is mainly due to the low tensile damage strength and the fact that tensile damage is brittle, which can lead to a higher load at the crack initiation point.

#### 3.2.3. Acoustic Emission

The acoustic emission [47] technique is used to monitor cracks and can infer crack closure, extension and spreading. Changes in the acoustic emission signal are important indicators for analyzing the evolution of damage within the structure during rock cracking and destruction. In this study, the acoustic emission characteristics under tensile loading after water chemical solution erosion were tested and analyzed. The number of acoustic emission events (referred to as the ringing AE event number) and the cumulative acoustic emission ringing counts, which indicate the degree of damage, were selected for investigation. The intrinsic relationship between the acoustic emission signals and rock damage is explored by analyzing the change pattern over time.

**Figure 11:**
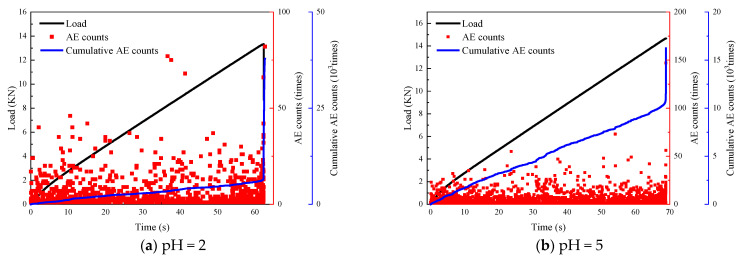

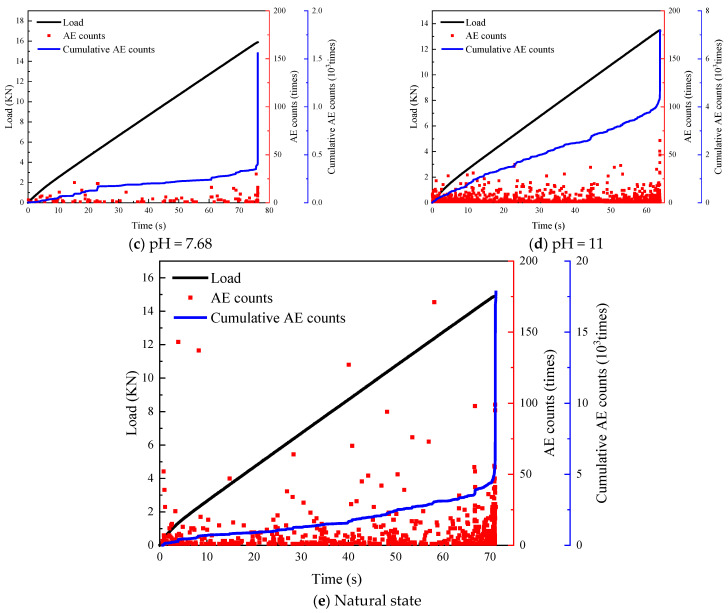
Acoustic emission ringing counts.

From Figure 11, the characteristic pattern of acoustic emission signals of sandstone specimens soaked in chemical solutions is consistent with that of the natural state. However, the difference lies in the fact that, during the compression and densification stage, the acoustic emission activity of the sandstone soaked in chemical solutions is more active compared to that of the sandstone samples in their natural state. This can be attributed to the weakening of particle cementation after the sandstone samples were immersed in the chemical solution. This weakening results in increased internal pore fissures, expansion of primary fissures and slight changes in the internal pore structure. The closure of internal primary pores and the slip friction between particles contribute to strong acoustic emission signals during the compaction stage.

During the loading process, the acoustic emission energy remains relatively stable and at a low level until the force value reaches its peak. This indicates that the initiation and development of cracks within the sandstone are relatively stable, and the release of elastic energy from these cracks is minimal. As the load slowly increases, the specimen approaches destruction when the external load approaches peak stress. At this point, there is a significant and sudden increase in the number of acoustic emission events, which corresponds to the rapid increase in the number of acoustic emission events. The elastic potential energy accumulated within the rock far exceeds the energy required to maintain the stable expansion of the cracks. Large and small cracks continue to expand and swell, forming a large number of macroscopic damage surfaces of different sizes. This leads to an immediate release of the accumulated elastic potential energy and the rock sample splits and is destroyed.

The acidity and alkalinity of the aqueous chemical solution have a significant effect on the cumulative number of AE events in the rock damage process. The acoustic emission signal becomes more intense with increasing acidity and alkalinity. The acoustic emission signal is more intense in the specimens corroded by strong acids and bases. This is due to the gradual expansion and development of primary pores inside the sandstone specimens after corrosion by aqueous chemical solutions. In the specimen, the chemical reaction of the product gradually forms a new chemical cementation bond. Therefore, during the loading process, the internal primary pore expansion rate is accelerated, and the probability of internal particle friction and crystal breakage events increases dramatically. The acoustic emission signal is more active during the whole loading process than in the natural state.

A similar pattern of agreement was obtained within the literature [48]: chemical corrosion leads to an increase in the number of tiny pores and fissures in the rock. During the subsequent compressive and tensile damage, the ringing counts in the compaction phase of the rock were significantly different, characterizing the different degrees of chemical corrosion. Rocks more severely chemically corroded have more ringing counts in the compaction phase than in the natural state.

## 4. Damage Mechanisms

### 4.1. Corrosion Mechanism Study

Dynamic measurements were conducted at various time intervals during the immersion process to monitor the pH values of the different chemical solutions used. The obtained data revealed a trend in the pH values as a function of chemical corrosion time. As the chemical corrosion time increases, all solution pH values converge to the middle value and gradually stabilize. Ultimately, the pH of the solution stabilizes at a weakly alkaline level (pH > 7.0). This is mainly due to the reduced concentration of H^+^ ions in the chemical solution, coupled with the alkaline nature of the chosen substrate solution. To mathematically describe the gradual stabilization of pH values over time in sandstone immersed in chemical solutions, the Exponential Decay function (abbreviated as “EXPDec”) was employed:

(2) $$pH=a\times e^{-bt}+c$$

The function successfully fits a nonlinear exponential decay function to pH monitoring data collected during the soaking process. Using this function, the gradual stabilization trend observed in sandstones immersed in chemical solutions can be effectively illustrated. The nonlinear exponential decay function was fitted to the pH monitoring data under different pH conditions. The parameters of the functional equation for the fitted curves for each pH condition are shown in Table 4.

Where a represents the size of the pH value in the initial state; *b* represents the rate of decay of the pH index, i.e., the rate at which the pH value decreases exponentially with the increase of the immersion time t; and c represents the minimum value of the pH value, i.e., the limiting value of the decay of the pH index of the solution. Typically, when the immersion time approaches infinity, the pH will tend to *c* value.

**Figure 12:**
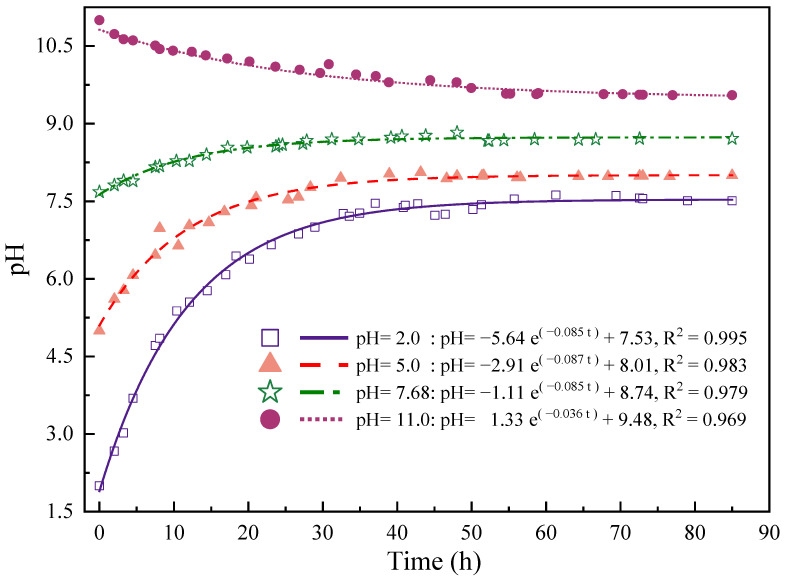
Change curve of solution pH during immersion in different pH solutions.

The rate and magnitude of change in pH of the sandstone leach solution at different pH solutions are shown in Figure 12. The pH index decay function of the exponential level of the rate of decrease is different, in which the relative magnitude of the rate of decay is strong alkaline (pH = 11.0) < strong acidic (pH = 2.0) < weakly acidic (pH = 5.0) < weakly alkaline (pH = 7.68); the change of the solution pH within 30 h. The relative magnitude of the changes in solution pH within 30 h was strongly acidic (pH = 2.0) > weakly acidic (pH = 5.0) > strongly basic (pH = 11.0) > weakly basic (pH = 7.68). Among them, under the environment of different pH solutions, different sandstone compositions and reaction rates of dissolution reaction lead to different pH and pH change rates of the final solution.

Carbonates are not resistant to acid solutions [49]. The dissolution rate of feldspar and calcite in sandstone in an acidic solution is inversely related to the pH value, i.e., the smaller the pH value, the faster the dissolution rate. Under acidic conditions, the concentration of H^+^ ions increases, making the calcite mineral, a constituent of sandstone, easy to react chemically with H^+^ under acidic conditions, for:

CaCO_3_(Calcite) + 2H^+^ → Ca^+^ + H_2_O + CO_2_↑ (3)

There is also a small amount of feldspar in the sandstone that reacts with H^+^ as well:

KAlSi_3_O_8_ + 4H^+^ + 4H_2_O → K + Al^3+^ + 3H_4_SiO_4_ (4)

Therefore, as time passes, the concentration of H^+^ ions decreases and the pH value of the solution increases and stabilizes.

Calcite is not easily dissolved in alkaline conditions and only a small amount of hydrolysis reaction exists. Only quartz in sandstone reacts readily in alkaline environments for:

SiO_2_ + 2OH^−^ → SiO_3_^2−^ + H_2_O (5)

These reaction processes consume OH^−^ ions, and all of these reactions result in a corresponding decrease in the solution OH^−^ ion concentration.

In a weak base (pH = 7.68) solution, the 2HCO_3_^−^ ions produced by the gradual dissolution of calcite gradually increase the pH of the solution until the dissolution is completely finished. Feldspar reacts at a slower rate under weakly alkaline conditions, which has a lesser effect on the pH of the solution. Therefore, as the concentration of H^+^ ions in the solution decreases over time, the pH will continue to rise until a stable pH is reached.

In a strong base (pH = 11) solution, the pH of the solution gradually decreases from 11 to 9.48, with the rate of change gradually slowing down. The chemical composition of the sandstone sample dissolves over time, resulting in a gradual decrease in the pH of the solution. As the reaction continues, the reaction rate gradually slows down, and the rate of change of the solution pH slows down accordingly.

### 4.2. Damage Variables

The generation and development of cracks within sandstone samples can be described by damage variables. Damage variables can be characterized by the acoustic emission characteristic parameters of the material, such as energy and ringing counts, etc. Scholars have given consistent proof of the damage variables of the rock and the acoustic emission cumulative ringing count. The damage of the material is manifested as the deterioration of material stress performance. This paper characterizes the damage mechanism through the ringing counts and cumulative ringing counts law under chemical corrosion with different pH values. Assuming that the sandstone is damaged completely (D = 1), an equivalent relationship between sandstone damage and the cumulative acoustic emission ringing counts is established:

(6) $$D=\frac{N}{N_{m}}$$

where *N_m_* is the cumulative count of acoustic emission when the whole cross-section of the rock sample is completely damaged; *N* is the cumulative count of acoustic emission during the damage process.

The damage to the rock is a process of continuous destruction and development of the microelement body. Assuming that the strength of the rock microelement conforms to the Weibull distribution, the damage variable of the rock under Brazilian splitting conditions can be expressed as:

(7) $$D=1-\exp[-{\left(\frac{\varepsilon }{n}\right)}^{m}]$$

where *m* and *n* are statistical parameters of the shape of the Weibull distribution.

**Figure 13:**
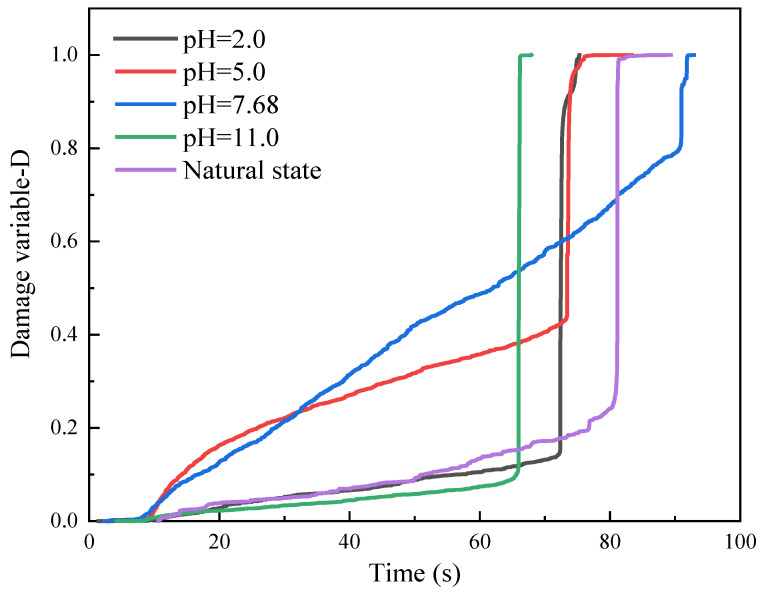
Damage variables.

Figure 13 represents the evolution curves of damage caused by chemical corrosion and loading at different pH values. The damage curves under the action of different pH chemical solutions differ greatly in time. In the initial stage of Brazilian splitting action, a large number of pores produced by chemical corrosion were compacted. The duration of the compacting stage was greater for weak bases than for strong bases, and greater for weak acids than for strong acids. This is most likely because the rock samples under strong acid and a strong base were damaged to a greater extent.

Based on the images, it can be seen that after the compaction stage, the damage curve increases rapidly to the highest point and the damage variable accumulates rapidly to 1.0. Since the rock is brittle and destructive, the images show a steep damage evolution curve in the crack extension stage. The overall chemical corrosion is small and enters the plastic deformation stage as the load increases. Microcracks penetrate to form large cracks. Eventually, the rock sample is damaged by splitting. This result indicates that the deformation of the rock increases due to the damage of the internal structure of the rock caused by chemical corrosion. It is feasible to use cumulative ringing counts as a chemical damage variable to quantitatively describe the degree of sandstone damage. This destruction law is consistent with the acoustic emission counting law. However, the correlation of the destruction variable with the time law is weaker. The correlation with the displacement law is stronger compared to previous studies [50,51,52], which can be strengthened in subsequent studies.

## 5. Conclusions

(1) The average tensile strength and splitting tensile modulus of the overall rock samples decreased gradually with the polarization of the solution pH. The highest point of the curve threshold is weakly alkaline. The decrease in tensile strength under acidic conditions is greater than the decrease in tensile strength under alkaline conditions.
(2) The solution pH on the sandstone Brazilian splitting compaction point will have an impact. As pH increases, the sandstone compaction point and peak load ratio is smaller, but the compaction load for the peak load is between 22% and 36%.
(3) The chemically eroded sandstone is characterized by an increase in acoustic emission ringing counts during the compaction phase. The rock samples, after chemical corrosion, showed a short duration of the compaction phase in the damage variable study and a steep damage evolution curve in the crack extension phase, with a rapid accumulation of the damage variable up to 1.0. These features suggest that chemical corrosion leads to an increase in pore fissures and deterioration of the internal structure.

## Figures and Tables

**Table 1:** Research perspectives of chemical corrosion.

Research Focus	Main Points
Degree of pH influence	The effect of pH on the uniaxial mechanical properties of sandstone is greater than the concentration [11,12].
pH saline solutions and damage patterns	The brine solution has some softening effects on the rock but has little effect on the rock damage pattern [8].
pH of acids and bases	The reduction in strength under acidic conditions is greater than that under alkaline conditions [13]. The peak strength of granite specimens under alkaline conditions shows varying degrees of intensification [14].
Salt effect	The dissolution rates in Na_2_SO_4_, Na_2_CO_3_ and NaCl solutions are all greater than their dissolution rates in pure (distilled) water, indicating that the salt effect has a significant impact on rock dissolution [15].
The same ion effect: the greater	The concentration of calcium and magnesium ions, preventing the dissolution of minerals, directly determines the rate and intensity of the dissolution of rock samples [16].
Ion concentration	The higher the concentration of dissolved ions in the solution, the greater the decrease in the mechanical parameters of the sandstone [17].

**Table 2:** Acoustic emission equipment parameters.

Main Frequency	Filter	Sampling Frequency	Parameters/Waveform Thresholds	Interval Parameter	Sampling Point	Trigger Mode
120 kHz	20–100 kHz	1000 kHz	40 DB	50 μs	2048 kHz	Internal trigger

**Table 3:** Specimen immersion.

Specimen	Diameter (mm)	Height (mm)	Mass (g)	Density (g/cm^3^)	Wave Velocity (m/s)	pH
DA-1	49.20	25.20	124.55	2.60	21.73	2.0
DA-2	50.06	25.05	128.10	2.60	21.73	
DA-3	50.00	25.04	128.06	2.60	21.60	
DB-1	49.99	25.05	127.82	2.60	21.73	5.0
DB-2	50.01	25.03	128.01	2.60	21.73	
DB-3	50.01	25.03	128.01	2.60	21.73	
DC-1	50.10	25.02	129.49	2.63	21.73	7.68
DC-2	49.20	25.20	124.34	2.60	21.73	
DC-3	49.98	25.04	127.95	2.60	21.73	
DD-1	50.00	25.03	129.42	2.63	21.73	11
DD-2	50.01	25.03	128.19	2.61	21.73	
DD-3	49.99	25.06	128.02	2.60	21.73	
D0-1	50.00	25.01	128.02	2.61	21.73	Distilled water
D0-2	50.01	25.01	128.06	2.61	21.73	
D0-3	50.10	25.02	129.00	2.62	21.73	

**Table 4:** Fitting model.

Immersion Solution pH	pH = 2.0	pH = 5.0	pH = 7.68	pH = 11.0
c	7.53	8.01	8.74	9.48
a	−5.64	−2.91	−1.11	1.33
*b*	0.085	0.087	0.085	0.036
R^2^(COD)	0.995	0.983	0.979	0.969

## Data Availability

The data used to support the findings of this study are available from the corresponding author upon request.
